# Health Care Professionals’ Social Media Behavior and the Underlying Factors of Social Media Adoption and Use: Quantitative Study

**DOI:** 10.2196/12035

**Published:** 2018-11-07

**Authors:** Joe Hazzam, Abdelmounaim Lahrech

**Affiliations:** 1 Faculty of Business and Law The British University in Dubai Dubai United Arab Emirates

**Keywords:** social media, health care professionals and social media, integrated behavioral model, technology acceptance theories

## Abstract

**Background:**

In the last decade, social media has emerged as a newer platform for knowledge dissemination, information exchange, and interpersonal communication for health care professionals (HCPs). However, the underlying behaviors of HCPs and the ethical use of social media for productivity enhancement and a sustainable health care system remain ambiguous.

**Objective:**

This study seeks to understand the factors that relate to the frequency use of social media in the health care discipline. It also aims to explore the underlying online behaviors of HCPs, which include the exchange of medical information with peers, interpersonal communication, and productivity enhancement in their daily practice.

**Methods:**

This study adopted the quantitative method in collecting and analyzing data. A survey instrument based on the behavioral and technology acceptance theories was developed for this purpose. The survey was distributed via social media platforms to 973 participants that included physicians, pharmacists, and allied HCPs working in the United Arab Emirates. The responses from 203 completed questionnaires (response rate 20.3%) were analyzed.

**Results:**

Of 203 respondents, 133 HCPs used WhatsApp (65.5%); therefore, WhatsApp had the highest number of users compared to Facebook and YouTube, with 101 users out of 203 (49.7%). Overall, 109 of 203 (53.6%) HCPs used social media platforms for the exchange of peer medical information and 108 of 203 (53.2%) used social media several times during the day to improve their interpersonal communication with colleagues. However, only 71 of 203 (34.9%) utilized social media to enhance their productivity in general. The structural model equation showed that behavioral intention (beta=.47; *P*<.001), habit (beta=.26; *P*=.001), attitude (beta=.20; *P*=.002), and perceived usefulness (beta=.12; *P*=.09) were positively and significantly related to frequency of use. The model explained a rate of 45% variance in the frequency of use and a rate of 17% variance in the social media intention of use.

**Conclusions:**

The research highlights the significant factors that relate to the adoption of social media platforms in health care practice. Based on the findings of this study, the use of online platforms facilitates the exchange of medical information among peers and enhances the share of experiences that support HCP’s learning and development. Moreover, social media platforms foster a higher level of communication among practitioners and might improve daily productivity. Future researchers might explore other variables such as training and external factors. For instance, they may draw on areas related to guidelines and policies. From this standpoint, the health care discipline can benefit from highly interactive platforms and adopt them for development, collaboration, and better health outcomes.

## Introduction

### Overview

With the advent of the internet, the health care sector—like any other discipline—has dramatically improved in terms of the speed of information exchange and the introduction of newer communication media. In fact, this advance in technology supports practitioners in developing better skills to yield better health outcomes and inform behavioral changes. The introduction of eHealth heralds a new era in developing advanced and innovative strategies to support the decision-making process in the health care sector. The advance of online technologies will also support practitioners in collecting, managing, and interacting for higher quality service. The growing use of social media platforms in the health care disciplines might also inform health behaviors in real time and support the exchange of information for better clinical outcomes [1]. They also support health campaign programs and patient education and provide an opportunity for information exchange and expansion of physician networks. However, poor quality of information, patient confidentiality, and legal issues are some risks and challenges that could impact the effective and beneficial integration of online platforms [2].

Social media usage differs in various health care settings because of the disparity between different medical tasks and responsibilities. Researchers have started to explore the rate of social media adoption and usability from the point of view of different stakeholders. In medical education, a study found that the degree of popularity and awareness is higher in undergraduate than in postgraduate circles. However, the study revealed that both groups had an interest in the use of new technologies [3]. Other researchers have studied the rate and usage of social media in different health care populations. For instance, pharmacists’ excessive use of social media is for the purpose of expanding their network and keeping in touch with old friends rather than for the purpose of education or professional development [4]. These findings are not in line with physician’s behaviors using social media. Adopting online platforms by doctors is increasing; however, guidelines and policies may act as barriers and reduce the quantity of information posted or shared in an effort to protect patient confidentiality [5]. The new online media are changing the communication behaviors of various stakeholders and increasing the exchange of information among health care professionals (HCPs) for an optimal decision process. However, the value of patient privacy should be respected on highly interactive and open platforms [6].

The main factor that affects the intention of professionals to participate in social media is the creation of virtual communities of practice. In fact, there is improved knowledge sharing among colleagues, but because these online groups are private, this confines knowledge to specific users and prevents the dissemination of information within a multidisciplinary environment to help improve performance and outcomes [7]. The use of social media within a health care context is affected by ethical dilemmas and privacy concerns that could prevent users from benefiting from this highly interactive means of communication. Research and trials are trying to explain the mechanism of information transfer and interaction by different users [8]. Practitioners in different health care disciplines can benefit from the significant data generated from the patients’ interaction within the social media groups. Moreover, interpersonal communication can be improved and evidence-based knowledge can be diffused faster than traditional channels. Research might explore the safe and the cost-effective use of social media to complement the evidence-based practice; moreover, the policies and guidelines can support the efficient adoption for personal development and knowledge updates [9].

The identification of the factors that promote practical use along with the effective type of platforms represents a gap in knowledge and requires further research [10]. The preliminary evidence of adopting social media in different health care practices reveals divergent opinions on the benefits, challenges, level of information exchange, communication, and productivity achieved. The study of users’ characteristics, behaviors, and external factors that relate to usage intention and frequency of use represent the potential to advance knowledge on the optimal integration of social media in health care practice. Additionally, it supports the extension of technology and information acceptance theories in the highly sophisticated settings of health care [11].

The primary research question is to understand the relationships between the influential factors and the adoption and usage of social media by HCPs. The research also aims to understand the strength of relationships between the underlying factors and the adoption of social media by HCPs. It also seeks to investigate the type and the frequency use of platforms by HCPs.

This research first attempts to define the theoretical research background. Next, it discusses, through a literature review, the relationships between the essential factors, the intention of social media adoption, and the actual use. Then, it presents the proposed conceptual model and the pilot study to test this conceptual model.

### Theoretical Background

The primary goal of the research is to understand the underlying behaviors, norms, and control factors that influence the intention and usage of social media by HCPs. The research proposes the presence of considerable complexities within a regulated and highly ethical discipline. Therefore, the study of the factors that inhibit or enhance the adoption of online platforms could inform future research to explore the intervention strategies that support active usage designed to improve health outcomes.

The theories of sociology, behavior change, and psychology are utilized to understand the acceptance of technology and usage in different organizational and consumer contexts. The Technology Acceptance Model (TAM) is adopted as a theoretical background due to the high level of prediction in adopting new technology [12]. The health care discipline is a highly regulated behavioral discipline that is governed by policies and guidelines. Therefore, the constructs of the Integrated Behavioral Model [13] will support the model and could inform future research to explore the intervention strategies that support active use for improving health outcomes. The constructs of the TAM emphasize the role of perceived usefulness and ease of use as main predictors of user behavior [12]. The TAM is identified as a theoretical background for the study of the perceived usefulness and ease of use as significant predictors of using social media technologies by two specialties of physicians in sharing and receiving information [14]. The TAM and the behavioral theories of reasoned actions [15] and planned behaviors [16] are employed as the theoretical background to study the acceptance of the personal digital assistant by HCPs for practical tasks achievements and easy access to medical records [17]. The research investigates the crucial factors that relate to the user’s behavioral intention. The professional image of practitioners within their social network influences their intention to use new technologies in the belief that a new system might enhance their image and social status. Therefore, the image of professionals is integrated into the research model as an important factor in the health care discipline. The acceptance of Web 2.0 tools for information sharing and interaction between nurses underlies the correlation between the independent variables of the behavior theories and the adoption of new technologies [18].

### Research Model and Hypothesis Development

Health care professionals face daily challenges and issues that entail a high level of communication and information exchange among colleagues. With the addition of social media, the decision-making process will be enhanced and will yield useful health outcomes and contribute to delivering outstanding performance. Public health professionals share a positive attitude toward the usage of online media for informing health change behaviors [19]. The HCPs communicate continuously to improve the performance of their daily tasks. Their use of Twitter has positively impacted their productivity and professional development. In fact, social media platforms are positively perceived for being an effective communication tool involving different stakeholders and for promoting further education and professional gain [20]. The dissemination of information and the update of knowledge are essential predictors of practitioners’ daily performance; therefore, social media enhances the processes involved in decision making. For example, dentists exhibit a positive attitude toward participation in social media platforms such as blogs and podcasts. They stress the practical role of online channels in sharing clinical outcomes and other relevant information, such as treatment options, that enhance the participants’ practical knowledge [21]. Health care professionals can analyze the content of the discussions and identify the best solution. This understanding will support the decision process and enhance patient awareness about the changes required [22]. The social media strategy of using different online tools and platforms such as blogs and tweets supports the evidence-based dissemination of child and youth diagnosis and treatment options. Therefore, information exchange and an organized communication strategy support pediatrics HCP’s personal development and productivity [23].

The attitude toward social media benefits and perceived outcomes are important predictors of HCP’s use of social media platforms [14,24]. Hypothesis 1 states that attitude will positively relate to the frequency of social media use. Hypothesis 2 states that perceived usefulness will positively relate to the frequency of social media use.

Social media opens up an opportunity for practitioners to interact, share, and develop knowledge through communication. The accessibility of such platforms reinforces the spread and transfer of knowledge [25]. User-friendly platforms that support timely responses, easy access, and easy navigation enhance the knowledge exchange of HCPs. In addition, communication supports a faster achievement of tasks [26]. Easy access to social media is a facilitator for HCP’s intention of use [8,14,24]. Hypothesis 3 states that ease of use will positively relate to the behavioral intention of social media use. Hypothesis 4 states that ease of use will positively relate to perceived usefulness.

The use of social media in health care practice might be useful for medical information exchange and interpersonal communication with peers. However, confidentiality within a highly controlled discipline raises many questions about the efficient use of social media without undermining the HCPs professional status. Without privacy control to protect the personal information of HCPs, the professional relationships with the patients could be altered as well as the effective communication of their health behavior messages [26]. Health care professionals and students are open to integrating social media in teaching content; however, the legal concerns and the technical issues remain major challenges for users and nonusers of online platforms [27]. The eHealth platforms are effective in addressing the patient’s problems; however, the privacy of intervention, reliability, and security are the main inhibitors of daily usage [28]. Social networking sites are useful for public health campaigns, but the management of privacy and personal data remains a significant concern for practitioners [29]. Privacy challenges, ethical concerns, and access to platforms are the essential concerns of the HCP’s intention of using social media [8,18]. Hypothesis 5 states that perceived control will positively relate to the behavioral intention of social media use. Hypothesis 6 states that perceived control will positively relate to ease of use.

Collaboration between colleagues, departments, and other stakeholders is fundamental to the achievement of health outcomes. Social status and relational identity with peers affect the HCP’s reinforced or varied use of social media [30]. The majority of pharmacy teachers joined Facebook’s online platform at the request of their friends or family members. They expressed their need to be socially connected with others [31]. Doctor-patient interaction on social media platforms strongly affects knowledge, efficacy, and perceived outcomes [32]. Experts and key opinion leaders are well respected in health care communities; moreover, they are the source of knowledge and act as advisers for juniors, residents, and newly graduated HCPs. The participation of qualified HCPs in online forums evokes a higher engagement rate by the practitioners [33]. The interactions between HCPs increases factual knowledge for higher productivity. Also, the presence of experts and key opinion leaders facilitates higher participation rates [34]. Hypothesis 7 states that perceived norms will positively relate to the behavioral intention of social media use. Hypothesis 8 states that image will positively relate to the behavioral intention of social media use. Hypothesis 9 states that image will positively relate to the perceived usefulness. Hypothesis 10 states that perceived norms will positively relate to the image.

Environmental constraints can inhibit actual behavior despite the presence of the intention [13]. Legal policies are emphasized as a critical factor in not sharing clinical images and content on social media channels by health care students [27,35]. The perceived usefulness and social interaction are the primary motivators for HCPs to join a virtual community of practice; however, time limitations are regarded as a barrier against use [36]. Hypothesis 11 states that environmental constraints will negatively relate to the frequency of social media use.

Health care practitioners use social media in their private life but using it in the workplace is affected by many factors that could facilitate or inhibit such behavior. The activities of HCP communities on the social media platform Twitter reveal the differences between the group’s followers and their habits of engagement through the retweet metric [37]. Previous positive experiences support a repetitive behavior that may become a habit [13,14,38]. Hypothesis 12 states that habit will positively relate to the frequency of social media use.

**Figure 1:**
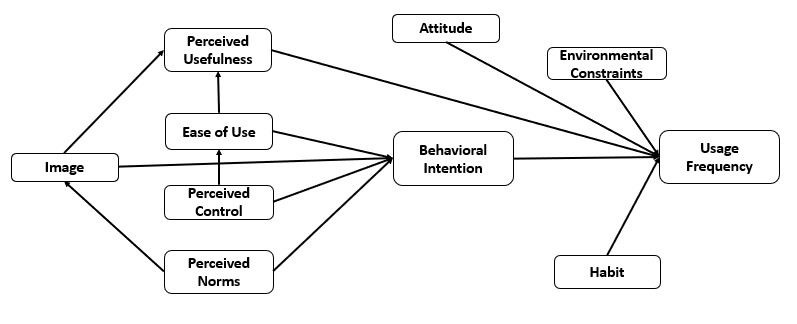
The research model.

The theory of reasoned actions, the theory of planned behaviors, and the technology acceptance theory explain that the best predictor of behavior change or the adoption of innovation is the intention toward the action [12,15,16,38]. The impact of behavioral intention of use has been tested empirically in different organizations and individual settings. Hypothesis 13 states that behavioral intention will positively relate to the frequency of social media use. Figure 1 demonstrates the proposed research model.

## Methods

### Measurement

Before designing the questionnaire, the researcher discussed the constructs of the proposed conceptual model with 10 experts in the field. The respondents in this elicitation phase [13] consisted of one dermatologist, one plastic surgeon, one dentist, one nurse, four pharmacists, one health insurance specialist, and one member from a hospital administration. Choosing different specialties during the design of the questionnaire was based on the researcher’s knowledge of the varied tasks and responsibilities within the health care context. Therefore, the opinions of the different stakeholders supported a holistic understanding of social media usage in health care practices.

The research model and expert validation informed the development of the study’s survey questions. The items and scales were adapted from previous researchers (see Multimedia Appendix 1). The questions about attitudes toward usage of social media were measured using a 10-point semantic scale adapted from McGowan et al [14]. The perceived usefulness, ease of use, and environmental constraint questions were adapted from Venkatesh et al [38], using a seven-point scale ranging from “strongly agree” to “strongly disagree.” The perceived norms, image, and perceived control questions were adapted from Yi et al [17] and measured as per the previous constructs scale. The questions about intention of use and the frequency of use were adapted from McGowan et al [14] and measured using a scale from “not aware” to “current user” and “never” to “many times daily,” respectively.

The questionnaire included 14 sections. The first section introduced the survey and consisted of the consent form. The second section concentrated on the demographics of the participants, including age, gender, specialty, years of experience, and organization type. The other parts concentrated on the 10 variables of the research model. Experts were consulted about the clarity of the questions and the appropriate time length needed for completing the survey.

### Data Collection

The three stratified target populations in this study were physicians, pharmacists, and allied employees working in any health care discipline located in the United Arab Emirates. The survey link was first sent via a LinkedIn account to the pharmacist population, which consisted of 518 participants. Next, the survey was administered to a WhatsApp group consisting of 235 medical doctors and 220 endodentists and other allied employees working in the United Arab Emirates. The survey generated 211 responses, with eight incomplete surveys or missing data. The number of completed questionnaires was 203, representing a response rate of 20.3% of the total survey views on the social media platforms of WhatsApp and LinkedIn.

### Data Analysis

Stata version 13.0 software was used in the data analysis of the research questions. The construct, convergent, and discriminant validity, as well as the reliability of the questionnaire items, were measured and explained. Confirmatory factor analysis emphasized the model fit. A structural equation model explained the relationships between the research model constructs.

## Results

### Participant Characteristics and Descriptive Statistics

Characteristics of participants are shown in Table 1.

**Figure 2:**
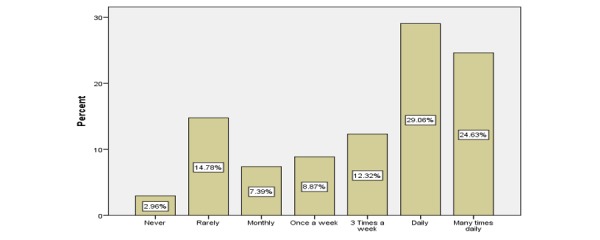
Frequency that the health care professionals used social media for the exchange of medical knowledge with peers.

**Figure 3:**
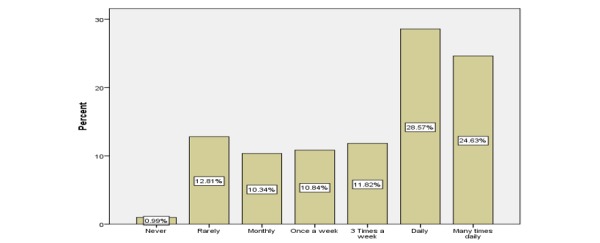
Frequency that the health care professionals used social media for improving their interpersonal communication with peers.

**Figure 4:**
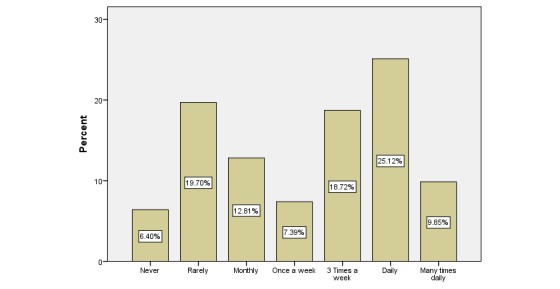
Frequency that the health care professionals used social media for increasing their overall productivity.

Of the 203 respondents, 145 (71.4%) were aged between 25 and 45 years and 130 (64.0%) were female HCPs. Most worked in the private sector (147/203, 72.4%). The sample included 101 (49.8%) physicians, 35 (17.2%) pharmacists, and 67 (33.0%) allied HCPs. The social media platform that had the highest current users was WhatsApp with 133 of 203 (65.5%), followed by Facebook and YouTube with 101 of 203 (49.7%). Of the 203 respondents, 109 (53.6%) HCPs used social media platforms for the exchange of medical information with their peers (Figure 2) and 108 of 203 (53.2%) used them several times a day to improve their interpersonal communication with colleagues (Figure 3). In addition, 71 of 203 respondents (34.9%) used these platforms to improve their overall productivity (Figure 4). The respondents’ areas of expertise correlated significantly with frequency of use (beta=.91, *P*=.02). Overall, 50 of 101 (49.5%) physicians, 15 of 35 (43%) pharmacists, and 43 of 67 (64%) allied employees used social media platforms for the exchange of medical knowledge with their peers. In addition, 47 of 101 (46.5%) physicians, 15 of 35 (43%) pharmacists, and 47 of 67 (70%) allied employees used social media platforms to improve their interpersonal communication with their peers. Finally, 31 of 101 (30.6%) physicians, 13 of 35 (37%) pharmacists, and 27 of 67 (40%) allied employees used social media to enhance their productivity in general.

**Table 1:** Participant characteristics (N=203).

Variable and category		Frequency, n (%)
**Age in years**		
	<25	35 (17.2)
	25-35	90 (44.3)
	36-45	55 (27.1)
	46-55	17 (8.4)
	56-65	5 (2.5)
	66-75	1 (0.5)
**Gender**		
	Male	73 (36)
	Female	130 (64)
**Type of organization**		
	Private	147 (72.4)
	Public	56 (27.6)
**Specialties**		
	Physician	101 (49.8)
	Pharmacist	35 (17.2)
	Allied	67 (33)

### Common Method Bias

The data were collected using the same questionnaire during the same period; therefore, the existence of common method bias was tested using the Harman single factor test which could inflate or deflate the estimates between the latent constructs [39]. Common method bias exists if only one factor explains 50% of the variance among the measures during the exploratory factor analysis with unrotated factor solutions [40]. In this study, the variance explained by the single factor was 24.88%, which is less than the 50% cutoff criteria, thus emphasizing the absence of common method bias.

### Measurement Model

Table 2 shows the results of the measurement model. To evaluate the construct validity and to confirm that the variable measurements represented what they were supposed to represent, an exploratory factor analysis was carried out on the 41 items [41]. The main purpose was to confirm that the questions of the survey reflected the constructs of the research model. Ten factors were extracted along with the items loaded on their intended constructs. Items BI1, BI3, and BI8 (see survey questions in Multimedia Appendix 1) were removed because their outer standardized loading was lower than .40 [42]. The study proceeded with reliability tests for the 10 factors included. Cronbach alpha for the total items was .891; hence, the score was higher than the recommended .7 [42]. Cronbach alpha for each factor extracted showed values higher than .7 (see Table 2). To assess the convergent validity, the composite reliability was calculated and the average variance extracted (AVE). The composite reliability should be greater than 0.70, and the AVE should be greater than 0.5 [43,44]. As shown in Table 2, the composite reliability and AVE values complied with these criteria.

The discriminant validity was evaluated using the Fornell-Larcker criterion [44]. The variance between the latent variables and their indicators should be higher than the variance explained with the other latent variables; therefore, the square root of the AVEs should be greater than the correlation between the constructs [44]. All diagonal square roots of the AVEs were greater than the correlation between the constructs represented in the off-diagonal elements (see Table 3).

**Table 2:** Cronbach alpha, composite reliability, and average variance extracted for the constructs.

Construct	Cronbach alpha	Composite reliability coefficient	Average variance extracted
Attitude	.87	0.90	0.60
Perceived usefulness	.85	0.84	0.57
Ease of use	.90	0.89	0.68
Image	.90	0.85	0.66
Perceived norms	.80	0.74	0.50
Perceived control	.89	0.87	0.65
Habit	.86	0.79	0.56
Environmental constraints	.72	0.83	0.63
Behavioral intention	.84	0.87	0.58
Use frequency	.86	0.82	0.61

**Table 3:** Correlations and the square roots of the average variance extracted. Off-diagonal elements are correlations. Diagonal elements are square roots of average variance extracted.

	AT^a^	PU^b^	EU^c^	IM^d^	PN^e^	PC^f^	HA^g^	EC^h^	BI^i^	UF^j^	Age	G^k^	P^l^	OT^m^
AT	.77													
PU	.12	.75												
EU	.11	.40	.82											
IM	.06	.48	.18	.81										
PN	.10	.55	.21	.57	.70									
PC	.10	.40	.29	.40	.35	.80								
HA	.10	.44	.48	.43	.48	.49	.74							
EC	.01	–.10	–.11	.05	–.07	–.16	.12	.79						
BI	.09	.20	.35	.01	.17	.13	.25	–.02	.76					
UF	.22	.33	.38	.19	.29	.35	.39	.09	.48	N/A^n^				
Age	–.04	–.03	–.28	.03	.04	–.05	–.18	.00	–.22	–.18	N/A			
G	.12	.02	–.08	.08	.12	.12	–.03	–.05	–.06	–.01	.23	N/A		
P	.08	.08	.13	.00	–.04	–.07	.06	.16	–.09	.17	–.04	–.15	N/A	
OT	.00	–.09	.00	–.30	–.20	–.12	–.04	–.04	–.05	.13	–.26	–.12	.03	N/A

^a^AT: attitude.

^b^PU: perceived usefulness.

^c^EU: ease of use.

^d^IM: image.

^e^PN: perceived norms.

^f^PC: perceived control.

^g^HA: habit.

^h^EC: environmental constraints.

^i^BI: behavioral intention.

^j^UF: use frequency.

^k^G: gender.

^l^P: profession.

^m^OT: organization type.

^n^N/A: not applicable because they were not reflective constructs.

### Structural Equation Modeling

**Figure 5:**
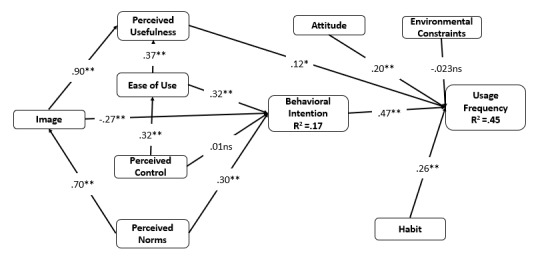
Structural model results. *P<.10; **P<.05; ns: nonsignificant.

In the first stage of structural equation modeling, the measurement model was specified and the goodness of fit was assessed. A covariance-based structural equation model was used to test the fit of the confirmatory factor analysis model to the research data [45]. The result of the confirmatory factor analysis for goodness of fit generated acceptable fit indexes (χ^2^_639_=1083.6, *P*<.001; confirmatory fit index [CFI]=0.907; root mean square error of approximation [RMSEA]=0.059). The second stage was the structural equation analysis. The indexes of the goodness of fit of the linear model were acceptable (χ^2^_614_=1094.7, *P*<.001; CFI=0.900; RMSEA=0.062]. The study also used path analysis and hypothesis testing. Table 4 presents a summary of the hypothesis statistical tests. Overall, the model explained 45% of the variance in the use frequency and 17% of the variance in the behavioral intention (see Figure 5). The predictors of the use frequency were the perceived usefulness (beta=.12, *P*=.09), the attitude (beta=.20, *P*=.002), the habit (beta=.26, *P*=.001), and the behavioral intention (beta=.47, *P*<.001). The predictors of the behavioral intention were the ease of use (beta=.32, *P*<.001), the image (beta=–.27, *P*=.02), and the norms (beta=.30, *P*=.02). To analyze if the ease of use mediated perceived control on behavioral intention, the indirect effect of perceived control on the behavioral intention was tested and was significant (beta=.09, *P*=.002), unlike the direct effect (beta=.01, *P*=.90). Therefore, it can be concluded that the ease of use mediated the relationship between perceived control and behavioral intention.

**Table 4:** Summary of findings regarding hypotheses.

Hypothesis	Beta	*z*	*P* value	Results
H1: The attitude will positively relate to the frequency of social media use	.20	3.15	.002	Accepted
H2: The perceived usefulness will positively relate to the frequency of social media use	.12	7.67	.09	Accepted
H3: The ease of use will positively relate to the behavioral intention of social media use	.32	4.42	<.001	Accepted
H4: The ease of use will positively relate to the perceived usefulness	.37	6.34	<.001	Accepted
H5: The perceived control will positively relate to the behavioral intention of social media use	.01	0.13	.99	Not accepted
H6: The perceived control will positively relate to ease of use	.32	5.26	<.001	Accepted
H7: The perceived norms will positively relate to the behavioral intention of social media use	.30	2.44	.02	Accepted
H8: The image will positively relate to behavioral intention of social media use	–.27	–2.35	.02	Not accepted
H9: The image will positively relate to the perceived usefulness	.90	9.66	<.001	Accepted
H10: The perceived norms will positively relate to the image	.70	13.64	<.001	Accepted
H11: The environmental constraints will negatively relate to the frequency of social media use	–.03	–0.51	.61	Not accepted
H12: The habit will positively relate to the frequency of social media use	.26	3.36	.001	Accepted
H13: The behavioral intention will positively relate to the frequency of social media use	.47	7.67	<.001	Accepted

## Discussion

### Principal Results

Health care professionals are exposed to an overwhelming amount of information that needs to be managed and applied effectively for task achievement. Social media platforms can foster a higher level of communication for knowledge and experience sharing. This study has explored the impact of different factors on social media use by HCPs. The frequency of use was measured through three main dimensions related to medical information exchange with peers, interpersonal communication, and productivity enhancement. The results reveal that the most used platforms are WhatsApp, Facebook, and YouTube. Previous research in a similar area concluded that HCPs use Twitter mostly for personal development and exchange of knowledge [46].

The results show that the frequency of use differs from one specialty to another. Such variation is explained by the users’ characteristics, the various tasks, and job responsibilities. The study confirms the role of attitude, perceived usefulness, habit, and behavioral intention as the major predictors of HCP’s frequency of social media use. The attitude variable was studied in previous research as the main factor that relates to the physicians’ frequency of social media use [14]. Moreover, behavioral intention is highly related to the frequency of use; hence, the theoretical construct of intention [12,13,15,16] was empirically tested in online settings within the HCP population. The results of this study support the role of habit in predicting the frequency of use, but the role of environmental constraints as an inhibitor of social media frequency of use cannot be confirmed [13]. The results may be explained by HCP’s characteristics as online users or the absence of policies regulating social media usage.

Ease of use positively relates to behavioral intention, which highlights easy access and navigation through social media platforms [23,24]. The norms predict the HCP’s intention of using the social media platforms and positively impact the image of the practitioners. The results reveal the positive role of the social network in the user level of engagement [47]. Moreover, the social relationships influence the participation of the HCPs in online Web-based platforms for better daily outcomes [33]. It was hypothesized that the image of HCPs would impact the behavioral intention of using social media platforms; however, this hypothesis remains unsupported.

The findings suggest that the image of HCPs relates negatively to the intention of use. Confidentiality, personal information, and the openness of social media platforms might explain such a negative relationship. Image highly predicts perceived usefulness. The findings also confirm that HCPs refer to experts or key opinion leaders for better decision processes and outcomes [34,48,49]. The perceived control does not relate significantly to behavioral intention. Rather, it positively and significantly relates to ease of use. The analysis of the indirect relationship between perceived control and behavioral intention is significant; therefore, the study confirms the mediation effect of ease of use.

### Limitations and Future Research

This research may be the first to explain variation in the frequency of use by different health care specialties. It also confirms the role of behavior and technology acceptance theories in predicting social media use by HCPs. However, there are limitations in the research. One is the convenience sample, which includes HCPs who are current users of social media platforms. The offline practitioners might provide a better understanding of the other important variables that influence frequency of use. The other is the absence of public health practitioners and researchers. Their presence would influence the perceived usefulness of the platforms since previous studies have dealt with cost reductions for recruiting patients through social media apps [50,51]. Future researchers might explore other variables such as training, for example. Hence, the health care discipline can benefit from the highly interactive platforms and employ it for the purpose of development, collaboration, and better health outcomes.

### Conclusion

New knowledge and experiences for HCPs are critical for career development. They need medical knowledge in real time to take decisions, as well effective communication with peers to resolve problems that arise in practice. Knowledge created and shared with colleagues and medical communities enhances performance and supports effective interventions for behavioral health changes. Social media platforms provide an opportunity for HCPs to interact, share information, and disseminate knowledge for better decision making. The importance of this study lies in the medical knowledge and practices shared by HCPs on social networks with their peers. This research suggests that the efficient use of social media platforms might significantly reduce the challenges raised within traditional health care settings. Furthermore, it can support practitioners’ career advancement by keeping them current and well-informed. This research adopts the theories of behavior changes and technology acceptance to build a model that can explain underlying user perceptions and other variables that contribute to the successful use of social media platforms by HCPs. The study model was tested, and the results confirm a rate of 45% variance in the frequency of use and 17% variance in the behavioral intention of using social media platforms. The attitude, perceived usefulness, behavioral intention, and habit significantly impact use frequency. The perceived norms, ease of use, and image have a considerable effect on behavioral intention. On this basis, this research has practical implications and efforts should be expended to create social media platforms that take into account the HCP’s characteristics, concerns, and objectives of use.

## Appendix

Multimedia Appendix 1 Survey instrument.

## Abbreviations

AVE: average variance extracted
CFI: confirmatory fit index
CR: composite reliability coefficient
HCP: health care professional
RMSEA: root mean square error of approximation
TAM: Technology Acceptance Model

## References

[1] Shaw T, McGregor D, Brunner M, Keep M, Janssen A, Barnet S (2017). What is eHealth (6)? Development of a conceptual model for eHealth: qualitative study with key informants. J Med Internet Res.

[2] Ventola CL (2014). Social media and health care professionals: benefits, risks, and best practices. P T.

[3] Sandars J, Schroter S (2007). Web 2.0 technologies for undergraduate and postgraduate medical education: an online survey. Postgrad Med J.

[4] Alkhateeb FM, Clauson KA, Latif DA (2011). Pharmacist use of social media. Int J Pharm Pract.

[5] von Muhlen M, Ohno-Machado L (2012). Reviewing social media use by clinicians. J Am Med Inform Assoc.

[6] Gholami-Kordkheili F, Wild V, Strech D (2013). The impact of social media on medical professionalism: a systematic qualitative review of challenges and opportunities. J Med Internet Res.

[7] Rolls K, Hansen M, Jackson D, Elliott D (2016). How health care professionals use social media to create virtual communities: an integrative review. J Med Internet Res.

[8] Dumas A, Lapointe A, Desroches S (2018). Users, uses, and effects of social media in dietetic practice: scoping review of the quantitative and qualitative evidence. J Med Internet Res.

[9] Grajales III IF, Sheps S, Ho KN, Novak-Lauscher H, Eysenbach G (2014). Social media: a review and tutorial of applications in medicine and health care. J Med Internet Res.

[10] Moorhead S, Hazlett D, Harrison L, Carroll J, Irwin A, Hoving C (2013). A new dimension of health care: systematic review of the uses, benefits, and limitations of social media for health communication. J Med Internet Res.

[11] Li J, Talaei-Khoei A, Seale H, Ray P, Macintyre CR (2013). Health care provider adoption of eHealth: systematic literature review. Interact J Med Res.

[12] Davis F (1989). Perceived usefulness, perceived ease of use, and user acceptance of information technology. MIS Quart.

[13] Fishbein M (2000). The role of theory in HIV prevention. AIDS Care.

[14] McGowan B, Wasko M, Vartabedian B, Miller R, Freiherr D, Abdolrasulnia M (2012). Understanding the factors that influence the adoption and meaningful use of social media by physicians to share medical information. J Med Internet Res.

[15] Fishbein M (1967). Readings in Attitude Theory and Measurement.

[16] Ajzen I (1991). The theory of planned behavior. Organ Behav Hum Dec.

[17] Yi M, Jackson J, Park J, Probst J (2006). Understanding information technology acceptance by individual professionals: Toward an integrative view. Inform Manage.

[18] Lau A (2011). Hospital-based nurses' perceptions of the adoption of Web 2.0 tools for knowledge sharing, learning, social interaction and the production of collective intelligence. J Med Internet Res.

[19] Franco M, Tursunbayeva A, Pagliari C (2016). Social media for e-Government in the public health sector: protocol for a systematic review. JMIR Res Protoc.

[20] Hart M, Stetten N, Islam S, Pizarro K (2017). Twitter and public health (Part 1): how individual public health professionals use Twitter for professional development. JMIR Public Health Surveill.

[21] Melkers J, Hicks D, Rosenblum S, Isett K, Elliott J (2017). Dental blogs, podcasts, and associated social media: descriptive mapping and analysis. J Med Internet Res.

[22] Merolli M, Gray K, Martin-Sanchez F, Lopez-Campos G (2015). Patient-reported outcomes and therapeutic affordances of social media: findings from a global online survey of people with chronic pain. J Med Internet Res.

[23] Dyson M, Newton A, Shave K, Featherstone R, Thomson D, Wingert A, Fernandes R, Hartling L (2017). Social media for the dissemination of Cochrane Child Health Evidence: evaluation study. J Med Internet Res.

[24] Roland D, Spurr J, Cabrera D (2017). Preliminary evidence for the emergence of a health care online community of practice: using a netnographic framework for Twitter hashtag analytics. J Med Internet Res.

[25] Demiris G (2006). The diffusion of virtual communities in health care: concepts and challenges. Patient Educ Couns.

[26] MacDonald J, Sohn S, Ellis P (2010). Privacy, professionalism and Facebook: a dilemma for young doctors. Med Educ.

[27] D'Souza K, Henningham L, Zou R, Huang J, O'Sullivan E, Last J, Ho K (2017). Attitudes of health professional educators toward the use of social media as a teaching tool: global cross-sectional study. JMIR Med Educ.

[28] Ariens L, Schussler-Raymakers F, Frima C, Flinterman A, Hamminga E, Arents B, Bruijnzeel-Koomen C, de Bruin-Weller MS, van Os-Medendorp H (2017). Barriers and facilitators to eHealth use in daily practice: perspectives of patients and professionals in dermatology. J Med Internet Res.

[29] Balatsoukas P, Kennedy C, Buchan I, Powell J, Ainsworth J (2015). The role of social network technologies in online health promotion: a narrative review of theoretical and empirical factors influencing intervention effectiveness. J Med Internet Res.

[30] Pan Z, Lu Y, Wang B, Chau P (2017). Who do you think you are? Common and differential effects of social self-identity on social media usage. J Manage Inform Syst.

[31] Metzger AH, Finley KN, Ulbrich TR, McAuley JW (2010). Pharmacy faculty members' perspectives on the student/faculty relationship in online social networks. Am J Pharm Educ.

[32] Wu T, Deng Z, Feng Z, Gaskin D, Zhang D, Wang R (2018). The effect of doctor-consumer interaction on social media on consumers' health behaviors: cross-sectional study. J Med Internet Res.

[33] Poirier J, Cobb N (2012). Social influence as a driver of engagement in a web-based health intervention. J Med Internet Res.

[34] Stewart S, Abidi S (2012). Applying social network analysis to understand the knowledge sharing behaviour of practitioners in a clinical online discussion forum. J Med Internet Res.

[35] O'Sullivan E, Cutts E, Kavikondala S, Salcedo A, D'Souza K, Hernandez-Torre M, Anderson C, Tiwari A, Ho K, Last J (2017). Social media in health science education: an international survey. JMIR Med Educ.

[36] Barnett S, Jones S, Bennett S, Iverson D, Bonney A (2013). Perceptions of family physician trainees and trainers regarding the usefulness of a virtual community of practice. J Med Internet Res.

[37] Mishori R, Singh L, Levy B, Newport C (2014). Mapping physician Twitter networks: describing how they work as a first step in understanding connectivity, information flow, and message diffusion. J Med Internet Res.

[38] Venkatesh, Thong, Xu (2012). Consumer acceptance and use of information technology: extending the unified theory of acceptance and use of technology. MIS Quart.

[39] Bagozzi R, Yi Y (1988). On the evaluation of structural equation models. JAMS.

[40] Podsakoff PM, MacKenzie SB, Podsakoff NP (2012). Sources of method bias in social science research and recommendations on how to control it. Annu Rev Psychol.

[41] Field A (2013). Discovering Statistics Using IBM SPSS Statistics. 4th edition.

[42] Churchill G (1979). A paradigm for developing better measures of marketing constructs. J Mark Res.

[43] MacKenzie S, Podsakoff P, Podsakoff N (2011). Construct measurement and validation procedures in MIS and behavioral research: integrating new and existing techniques. MIS Quart.

[44] Fornell C, Larcker D (1981). Structural equation models with unobservable variables and measurement error: algebra and statistics. J Mark Res.

[45] Anderson JC, Gerbing DW (1988). Structural equation modeling in practice: a review and recommended two-step approach. Psychol Bull.

[46] Alsobayel H (2016). Use of social media for professional development by health care professionals: a cross-sectional web-based survey. JMIR Med Educ.

[47] Centola D (2010). The spread of behavior in an online social network experiment. Science.

[48] Francke A, Smit M, de Veer AJ, Mistiaen P (2008). Factors influencing the implementation of clinical guidelines for health care professionals: a systematic meta-review. BMC Med Inform Decis Mak.

[49] Bennett NL, Casebeer LL, Zheng S, Kristofco R (2006). Information-seeking behaviors and reflective practice. J Contin Educ Health Prof.

[50] Davies B, Kotter M (2018). Lessons from recruitment to an internet-based survey for degenerative cervical myelopathy: comparison of free and fee-based methods. JMIR Research Protocols.

[51] Christensen T, Riis A, Hatch E, Wise L, Nielsen M, Rothman K, Toft Sørensen H, Mikkelsen E (2017). Costs and efficiency of online and offline recruitment methods: a web-based cohort study. J Med Internet Res.

