# Supplementary material for: Health Care Professionals’ Social Media Behavior and the Underlying Factors of Social Media Adoption and Use: Quantitative Study
Source: J Med Internet Res. 2018 Nov 7;20(11):e12035. doi: 10.2196/12035 (PMC6249501; doi:10.2196/12035)
Supplement: Multimedia Appendix 1 [file jmir_v20i11e12035_app1.pdf]

Multimedia Appendix I – Questionnaire items and scales.

Attitude (1-10; Semantic Differential)

AT1 Attitude toward the social media Waste of time......Essential use
AT2 Attitude toward the social media Very Risky........ Very Beneficial
AT3 Attitude toward the social media Boring ....engaging
AT4 Attitude toward the social media Bad Way......Great way for knowledge exchange
AT5 Attitude toward the social media Bad Way......Great way for interpersonal communication
AT6 Attitude toward the social media Bad Way......Great way for Improving my productivity

Perceived Usefulness (1-7; Strongly disagree to Strongly agree)

PU1 I find social media useful in my daily life
PU2 Using social media increases my productivity
PU3 Using social media improves my Interpersonal communication
PU4 Using social media supports the exchange of knowledge

Ease of Use (1-7; Strongly disagree to Strongly agree)

EU1 Learning how to use social media is easy for me
EU2 My Interaction with social media is clear and understandable
EU3 I find social media easy to use
EU4 It is easy for me to become skillful at using social media

Image (1-7; Strongly disagree to Strongly agree)

IM1 People in my practice setting who use social media have more prestige than those who do not.
IM2 People in my practice setting who use social media have a high profile
IM3 Using social media is a status symbol in my practice setting

Perceived Norms (1-7; Strongly disagree to Strongly agree)

PN1 People whose opinions I value prefer me to use social media
PN2 At work, my colleagues who are important to me think that i should use social media
PN3 People who are important to me think that i should use social media

Perceived Control (1-7; Strongly disagree to Strongly agree)

PC1 I am able to use social media in my work
PC2 I am able to use social media despite the legal concern
PC3 I am able to use social media despite the privacy concern
PC4 I am able to use social media despite the Ethical concern

Habit (1-7; Strongly disagree to Strongly agree)

HA1 The use of social media has become a habit for me
HA2 I am addicted to use social media
HA3 I must use social media

Environmental Constraints (1-7; Strongly disagree to Strongly agree)

EC1 I am too busy to participate in social media
EC2 I don't have time to use social media for increasing my productivity
EC3 I am concerned about work policies when using social media

Behavioral Intention (1-5; Not aware to current user)

BI1 Restricted online communities
BI2 Facebook
BI3 Twitter
BI4 Instagram
BI5 LinkedIn
BI6 WhatsApp
BI7 YouTube
BI8 Snapchat

Frequency of usage (1-7; Never to many times a day)

UF1 What is your overall frequency of using social media for knowledge exchange
UF2 What is your overall frequency of using social media for increasing your productivity
UF3 What is your overall frequency of using social media for interpersonal communication
